# Standardizing feeding strategies for preterm infants born greater than 1500 grams

**DOI:** 10.1038/s41390-024-03483-y

**Published:** 2024-08-17

**Authors:** Ting Ting Fu, Maame Arhin, Ashley T. Schulz, Abigail Gardiner, Stacie Chapman, Abigail Adamchak, Laura P. Ward, Jae H. Kim

**Affiliations:** 1 Perinatal Institute, Division of Neonatology, Cincinnati Children’s Hospital Medical Center, Cincinnati, OH USA; 2 University of Cincinnati College of Medicine, Cincinnati, OH USA; 3 Division of Neonatology, Children’s Hospital of Philadelphia, Philadelphia, PA USA; 4 Department of Pediatrics, Cincinnati Children’s Hospital Medical Center, Cincinnati, OH USA; 5 University of Cincinnati Medical Center, Compass One, Cincinnati, OH USA

## Abstract

**Background:**

Use of standardized feeding protocols and donor breast milk (DBM) have been studied primarily in infants born <1500 g and not examined exclusively in infants born >1500 g.

**Methods:**

In this retrospective pre-post-implementation cohort study, we evaluated a protocol for preterm infants born >1500 g that was implemented clinically to standardize feeding advancements at 30 mL/kg/day, with infants born <33 weeks eligible to receive DBM. We compared placement of peripherally inserted central catheters for parenteral nutrition, feeding tolerance, growth, and maternal milk provision in the 18 months before/after implementation. The association between DBM intake and growth was evaluated using multivariable linear regression.

**Results:**

We identified 133 and 148 eligible infants pre/post-implementation. Frequency of peripherally inserted central catheters and rate of maternal milk provision was not statistically different. While there was no difference in median days to full enteral volume, there was a narrower distribution post-implementation (*p* < 0.001). Growth was similar between eras, but each 10% increase in DBM was associated with 1.0 g/d decrease in weight velocity (*p* < 0.001).

**Conclusions:**

A feeding protocol for preterm infants >1500 g is associated with more consistent time to full enteral volume. Further investigation is needed to clarify DBM’s impact on growth in this population.

**Impact:**

- Despite practice creep, no study has examined the use of standardized feeding protocols or pasteurized donor breast milk exclusively in infants >1500 g.
- A feeding protocol in this population may achieve full enteral feedings more consistently.
- With appropriate fortification, donor breast milk can support adequate growth in infants born >1500 g but warrants further study.

## Introduction

Feeding strategies for preterm infants born greater than 1500 g have not been clearly evaluated. Existing nutritional strategies for neonates have largely focused on the higher risk population of very low birth weight (VLBW) infants (birth weight less than 1500 g).^1,2^ Two such strategies are standardized feeding protocols and the use of pasteurized donor breast milk (DBM) when maternal breast milk (MBM) is not available.^3–6^ It is unknown whether these evidence-based feeding strategies for VLBW infants can be generalized to infants born greater than 1500 g, who represent a significant proportion of neonatal admissions and whose risk of morbidity and mortality is not neglibile.^7^

Standardized feeding protocols are a consistent method to improve outcomes for VLBW infants.^8^ Benefits include shorter time to reach full enteral volume, reduction in the incidence of necrotizing enterocolitis (NEC), decreased variation in nutritional management, and improved growth.^3,9^ Faster attainment of full enteral feeding also decreases the duration of dependency on parenteral nutrition, which is not a benign intervention and can be associated with complications related to central venous catheters, including infection and extravasation.^10^ Without a standardized feeding protocol in place, medical providers may be inconsistent, with some still opting for slower feeding advancements and placement of a central venous catheter, despite evidence showing no difference in incidence of NEC or death with faster versus slower feeding advancements.^11,12^

One key component of standardized feeding protocols is the enteral feeding source, and pasteurized DBM is the recommended alternative for VLBW infants when MBM is unavailable.^13^ Presence of DBM in NICUs in the United States has increased in the last decade, but there is significant variation in the eligibility and duration of its use.^13–17^ Extending the provision of DBM to larger preterm infants who would otherwise receive preterm formula, either as supplementation to MBM or primary diet, offers them some of the unique benefits of human milk that currently cannot be mimicked otherwise, such as improved feeding tolerance, though it remains unclear whether there is a measurable clinical difference. However, concerns exist regarding the nutritional composition of DBM and its association with suboptimal growth outcomes.^18,19^ The evidence is also mixed whether the provision of MBM is affected by DBM availability.^20–23^ It is not known whether these concerns persist beyond the VLBW population.

Thus, we aim to assess the utilization of a standardized feeding protocol and DBM in preterm infants born greater than 1500 g to compare placement of a central catheter for nutrition, days to full enteral feeding volume, and growth metrics before and after implementation. We hypothesize that the combination of a feeding protocol with DBM availability will reduce the necessity for a central venous line to support slower feeding advancements without negatively impacting growth.

## Methods

This retrospective cohort study was conducted at a level III NICU in Cincinnati, Ohio, and was approved by the Cincinnati Children’s Hospital Institutional Review Board with a waiver of authorization and consent (#2020-0801).

In January 2019, to standardize practice and reduce variation in the placement of a peripherally inserted central catheters (PICC) for nutrition and achieve goal enteral feeding volume faster, the NICU implemented a feeding protocol for preterm infants born less than 34 weeks completed gestation and with birth weight greater than 1500 g. At the same time, because of practice creep to allow DBM to be available beyond VLBW infants, such as those born at 31–32 weeks, the eligibility for DBM was increased from VLBW to include all infants born less than 33 weeks completed gestation. This feeding protocol was developed as a corollary to a well-established standardized feeding protocol for VLBW infants that requires placement of a PICC for total parenteral nutrition and has been previously described.^24,25^ In brief, for the >1500 g protocol, enteral feedings are initiated in clinically stable infants within 24 h of life at 20 mL/kg/day, then advanced by 30 mL/kg/day in two steps (15 mL/kg/day every 12 h). After tolerating 110 mL/kg/day of enteral feedings, unfortified human milk is fortified directly to 24 kcal/oz with Similac Human Milk Fortifier Extensively Hydrolyzed Protein Concentrated Liquid (Abbott Nutrition, Abbott Park, IL). Subsequently, feedings are advanced by 20 mL/kg/day in two steps (10 mL/kg/day every 12 h) until goal. See Appendix 1 for more details of the >1500 g feeding protocol. A comparison of the steps of the VLBW and the >1500 g protocols is shown in Supplemental Table 1. Of note, due to the pace of the >1500 g protocol, a PICC is not required. Probiotics administration was not a part of unit practice during the study period.

For the study, infants born weighing more than 1500 g and less than 34 weeks gestational age were identified from the 18-month period prior to and after implementation of the >1500 g protocol. Infants who died or transferred in the first week of life were excluded. For all eligible infants, demographic and clinical information was collected from the medical chart, including feeding protocol selection, placement of a PICC for nutrition (primary outcome), length of stay, days receiving parenteral nutrition or intravenous fluids, late onset sepsis, and diagnosis of NEC (any Bell’s stage). Nutritional data of interest included days to achieve full enteral volume, first feeding substrate, highest caloric density for fortification, whether each infant received any MBM or was receiving MBM at discharge, and whether each infant ever directly breastfed or was directly breastfeeding at discharge. Because the total fluid goal typically ranges from 140–160 mL/kg/day in our unit, we defined full enteral volume as 140 mL/kg/day based on birth weight. DBM and MBM intake was calculated by dividing the total volume of DBM or MBM intake by the total enteral intake during the entire NICU stay. Anthropometric measurements obtained at birth, 28 days (if still hospitalized), and discharge were recorded and converted to Fenton z-scores.^26^ Body mass index (BMI) was calculated and converted to Olsen z-score.^27^ Growth velocities were calculated from birth to discharge and from birth to 28 days.

For statistical analysis, pre- and post-implementation clinical outcomes and growth metrics were compared by Chi-square, Fisher’s exact, Mann–Whitney U, and t-tests. Since DBM eligibility was limited to less than 33 weeks, infants born between 33 and 34 weeks gestational age were excluded for nutrition and growth analyses. Multivariable linear regression modeling was performed to examine the relationship between DBM intake and each growth outcome, adjusting for a priori selected confounders of gestational age, sex, and cohort era. These analyses were performed using SAS version 9.4 (SAS Institute Inc., Cary, NC). Additional comparison of distribution was performed using the two-sample Anderson-Darling test with R version 4.3.2 (R Core Team) and the goftest package (version 1.2-3).^28^ Results were considered statistically significant for *p* values < 0.05.

## Results

**Fig. 1:**
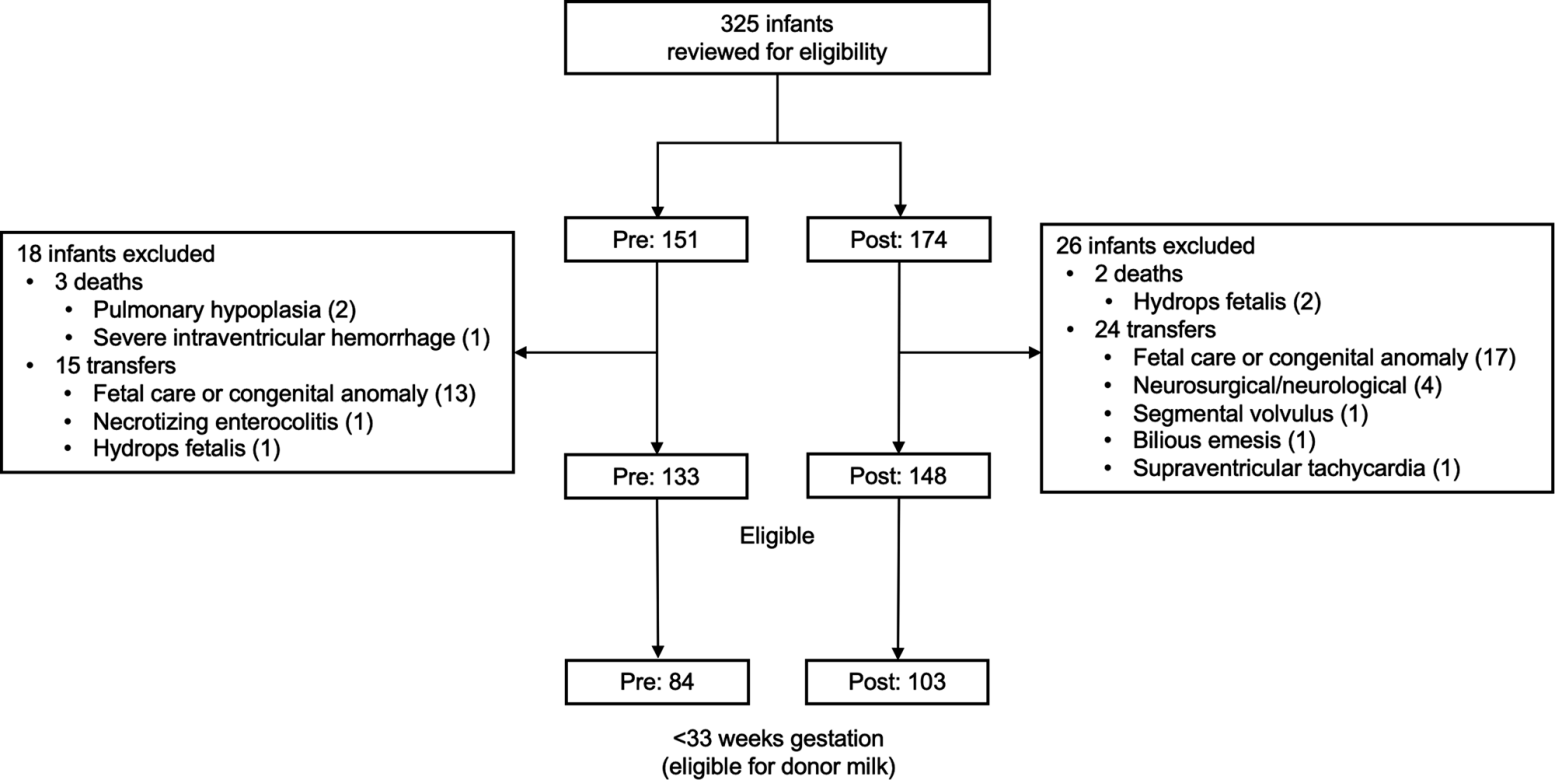
Flow diagram of patient inclusion and exclusion.

325 infants were reviewed for eligibility, and 133 and 148 infants were identified in the pre- and post-implementation eras respectively. Out of these, 84/133 and 103/148 were born prior to 33 weeks completed gestation and were eligible to receive donor milk. Figure 1 depicts the patient flow diagram of inclusion and exclusion. Of the 44 excluded infants, one case (in the pre-implementation cohort) was related to NEC (stage 3).

**Fig. 2:**
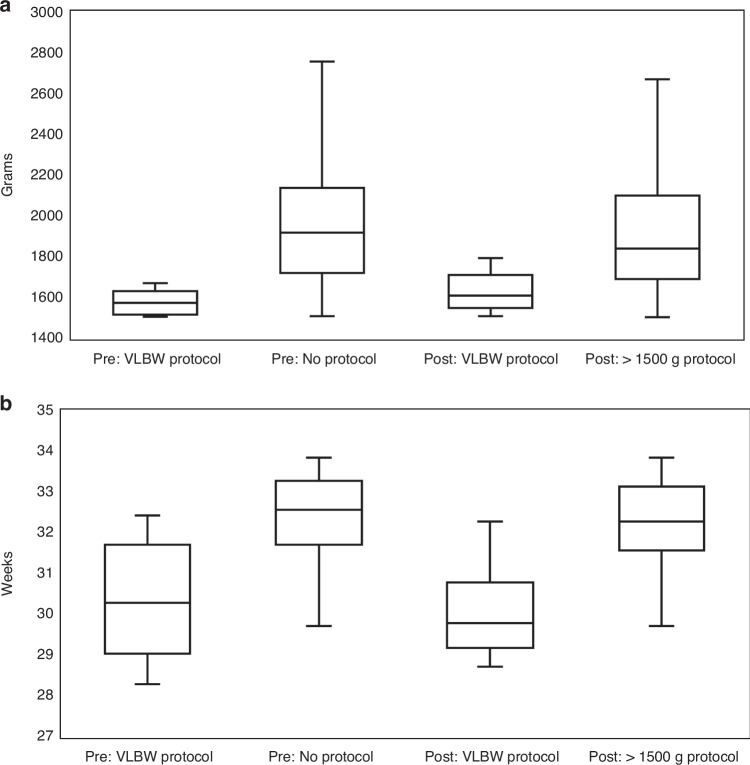
Distribution of infants by feeding protocol and era. Boxplots of infant (**a**) weight and (**b**) gestational age at birth, grouped by the feeding protocol the clinical team utilized, before/after implementation of the >1500 g Protocol. Pre (VLBW Protocol) *n* = 10, Pre (No Protocol) *n* = 123, Post (VLBW Protocol) *n* = 18, Post (>1500 g Protocol) *n* = 130. VLBW very low birth weight.

Table 1 shows the demographic and overall clinical outcomes, which were similar for the two groups. Compliance with initiation of the >1500 g feeding protocol in the post-implementation era was 88%, with 18/148 (12%) of infants still being placed on the slower VLBW feeding protocol and subsequently receiving a PICC for parenteral nutrition. Of the five cases of late onset sepsis in the post-implementation cohort, none were associated with the presence of a PICC. Incidence of NEC was similar between eras (for all cases or for only stage 2 and 3). Of the infants diagnosed with NEC, one patient from each era had received slower feeding advancements per the VLBW protocol. Characteristics of the 28 infants who received slower feeding advancements per the VLBW protocol (10 pre-implementation, 18 post-implementation) are described in Supplemental Table 2. There was no statistically significant difference between the eras in either birth weight (*p* *=* 0.21) or gestational age (*p* *=* 0.63) for these 28 infants (Fig. 2). However, in both eras, the range in birth weight and gestational age of infants placed on the slower VLBW protocol overlapped with those who were advanced faster (Fig. 2).

**Table 1:** Infant characteristics and clinical outcomes, before and after implementation.

	Pre (*n* = 133)	Post (*n* = 148)	*p* value
Birth weight (g)	1879.9 (1687.9–2118)	1795.1 (1668.1–2023.5)	0.16
Gestational age at birth (weeks)	32.4 (31.7–33.3)	32.1 (31.3–33.1)	0.11
Gestational age at discharge (weeks)	35.7 (35–36.7)	35.7 (34.9–36.9)	0.99
Sex (male)	74 (56%)	93 (63%)	0.22
Multiple gestation	0 (0%)	4 (3%)	0.12
Maternal gestational diabetes	24 (18%)	18 (12%)	0.17
Maternal preeclampsia	39 (29%)	46 (31%)	0.75
Small for gestational age	1 (1%)	0 (0%)	0.47
Length of stay (days)	24 (16–33)	24 (18–34)	0.27
Central venous line placement (any during admission)	19 (14%)	30 (20%)	0.19
Fed per VLBW feeding protocol / PICC placed for parenteral nutrition	10 (8%)	18 (12%)	0.19
Necrotizing enterocolitis (any Bell’s stage)	3 (2%)	5 (3%)	0.57
Necrotizing enterocolitis (stage 2 or 3)	1 (1%)	4 (3%)	0.37
Late onset sepsis	1 (1%)	5 (3%)	0.13
Days receiving parenteral nutrition or intravenous fluids	4.7 (3.3–5.9)	4.2 (3.2–5.5)	0.19

Median (interquartile range) or n (%).

*PICC* peripherally inserted central catheter, *VLBW* very low birth weight.

**Fig. 3:**
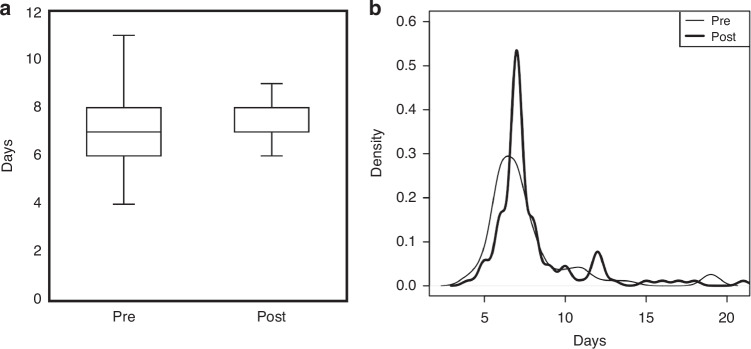
Days to full enteral volume feedings. **a** Boxplots (without outliers shown) and (**b**) density curves of days to full enteral volume feedings, before and after implementation. Pre *n* = 84, Post *n* = 103. Mann–Whitney U *p* = 0.06, Anderson-Darling two-sample *p* = 0.03.

Table 2 presents the nutrition outcomes between the two eras. Because of the extended eligibility of DBM post-implementation, there was a higher incidence of human milk at first feeding (68% pre vs. 90% post, *p* *<* 0.001) and percentage of DBM intake (1.3% pre vs. 13.6% post, *p* *=* 0.006). Median days to full enteral volume was not different (*p* *=* 0.06), but there was a narrower interquartile range post-implementation with statistically different distribution (*p* *=* 0.03) and a concentrated higher peak (Fig. 3). Rates of initiation or sustainment of either MBM expression or direct breastfeeding were not altered.

**Table 2:** Nutrition outcomes for infants born <33 weeks, before and after implementation.

	Pre (*n* = 84)	Post (*n* = 103)	*p* value
First feeding human milk	57 (68%)	93 (90%)	<0.001
Highest level of fortification			
24 kcal/oz	49 (58%)	56 (54%)	0.36
26 kcal/oz	33 (39%)	43 (42%)	
28 kcal/oz	2 (2%)	1 (1%)	
30 kcal/oz	0 (0%)	3 (3%)	
Days to full enteral feeding volume	7 (6–8)	7 (7–8)	0.06
% donor milk intake	1.3 (0–35.4)	13.6 (0.8–46.2)	0.006
% maternal milk intake	74.3 (17.2–97.7)	63.6 (24.1–97.1)	0.99
Mother initiated pumping	74 (88%)	84 (82%)	0.22
Receiving maternal milk at discharge^a^	46 (55%)	49 (49%)	0.43
Direct breastfed ever	48 (57%)	53 (51%)	0.44
Direct breastfed within 72 h of discharge*	20 (24%)	16 (16%)	0.18

Median (interquartile range) or n (%).

^a^Four infants transferred prior to discharge, pre *n* = 83, *n* = 99.

Table 3 shows the growth velocities and changes in z-scores over time, which were not statistically different before and after implementation. For infants whose length of stay was greater than 28 days, length velocity was modestly decreased in the first 28 days (1.1 cm/week pre vs. 0.9 cm/week post, *p* *=* 0.08) with the availability of DBM. For this subset of patients, this difference in linear growth was not observed out to discharge (1.0 ± 0.3 cm/week pre vs. 0.9 ± 0.3 cm/week post, *p* *=* 0.20). Multivariable regression analyses comparing donor milk intake to growth parameters are depicted in Table 4. After controlling for gestational age, biological sex, and cohort era, the most significant relationship was observed between DBM exposure and weight velocity, with every 10% increase in DBM intake being associated with a decrease in weight velocity of −1.01 g/day (95% CI −1.43, −0.58, *p* *<* 0.001).

**Table 3:** Growth outcomes for infants born <33 weeks, before and after implementation.

	Birth to discharge			Birth to 28 days		
	Pre (*n* = 84)	Post (*n* = 103)	*p* value	Pre (*n* = 38)	Post (*n* = 48)	*p* value
Weight velocity (g/day)	21.5 (8.9)	21.4 (8.7)	0.99	26.9 (5)	26.1 (6.4)	0.53
Length velocity (cm/week)	0.8 (0.5)	0.8 (0.6)	1.00	1.1 (0.4)	0.9 (0.4)	0.08
HC velocity (cm/week)	0.7 (0.4)	0.6 (0.4)	0.21	0.7 (0.3)	0.7 (0.3)	0.99
Change in weight z-score	−0.6 (0.4)	−0.7 (0.5)	0.43	−0.6 (0.4)	−0.6 (0.4)	0.96
Change in length z-score	−0.6 (0.6)	−0.7 (0.7)	0.61	−0.5 (0.7)	−0.7 (0.6)	0.14
Change in HC z-score	−0.3 (0.7)	−0.4 (0.8)	0.58	−0.5 (0.9)	−0.4 (0.9)	0.81
Change in BMI z-score	−0.4 (0.8)	−0.4 (1)	0.95	−0.4 (1.1)	−0.1 (0.9)	0.24

Mean (standard deviation).

*BMI* body mass index, *HC* head circumference.

**Table 4:** Multivariable regression models comparing donor milk exposure with growth outcomes.

	Beta Estimate (95% CI)	*p* value
Weight velocity (g/day)	−1.01 (−1.43, −0.58)	<0.001
Length velocity (cm/week)	−0.02 (−0.05, 0.01)	0.18
HC velocity (cm/week)	−0.01 (−0.03, 0.01)	0.57
Change in weight z-score	−0.03 (−0.06, −0.01)	0.004
Change in length z-score	-0.03 (−0.07, 0.01)	0.11
Change in HC z-score	−0.03 (−0.07, 0.01)	0.20
Change in BMI z-score	−0.01 (−0.06, 0.04)	0.79

Adjusted for gestational age, sex, and cohort era. Beta estimate reported per 10% increment in donor milk intake.

*BMI* body mass index, *CI* confidence interval, *HC* head circumference.

## Discussion

In this retrospective study, we demonstrated that implementation of a standardized feeding protocol for infants born greater than 1500 g was not associated with a change in the frequency of PICC placement for parenteral nutrition, our primary outcome. We did, however, achieve more consistency in the time to reach full enteral volume and observed no difference in growth trajectories after implementation, as secondary outcomes.

We selected PICC placement for parenteral nutrition as a primary outcome because the original goal behind the clinical implementation of the protocol in the first place was to minimize PICC placement by reaching full feeding volume faster. Central venous access allows for more concentrated parenteral nutrition delivery, but these catheters carry both infectious and non-infectious risks, such as migration and occlusion.^29–31^ Although there was not a statistically significant difference between cohort eras, we found 88% compliance with the protocol with a median of 4–5 days of intravenous fluids or parenteral nutrition and 7 days to full feeding volume. While we did not separate the two types of fluids, it may be that this subpopulation of preterm infants benefits only minimally from parenteral nutrition given the short duration of intravenous support. Similar to our cohort, most of the moderate and late preterm infants in the DIAMOND trial achieved full feeding volume within a week, and although there were some differences in short term growth between infants who received parenteral nutrition versus dextrose-only fluids, their body composition were similar at four months corrected age.^32^ In addition, early fortified or enriched enteral feedings can be an option to bridge nutrient intake if not utilizing parenteral nutrition.^25,33,34^

We suspect our observed 12% non-compliance was partly due to sustained perception from some members of the clinical team that smaller and younger preterm infants (for example, those born just above 1500 g) remain at increased risk for NEC and may benefit from slower feeding advancements, but this concern and subsequent non-compliance was inconsistent, as there was overlap in the gestational age and birth weight ranges of infants in each protocol group. Previous studies of standardized feeding protocols for preterm infants, irrespective of the exact protocol, described an association with a reduced risk for NEC, though the studies that included preterm infants up to 2000 or 2500 g were older (1978–2006) and did not separate VLBW from larger infants to provide stratified results.^3,35–39^ To our knowledge, our study is the first to evaluate the use of a standard feeding protocol exclusively in preterm infants who don’t fall under the higher risk VLBW categorization. We identified eight total cases of NEC in the >1500 g population in this three-year span (2.8%), consistent with the Neonatal Research Network.^7^ Although we observed that the incidence of NEC was unchanged after implementation, we suspect this was due to our low baseline rate. Importantly, standardized feeding advancements at 30 mL/kg/day (15 mL/kg/day every 12 h) was not associated with an increase in the incidence of NEC. This is consistent with a Cochrane systematic review and the SIFT trial, which both found no difference in incidence of NEC or death with faster (30–40 mL/kg/day) versus slower feeding advancements in VLBW and very preterm infants,^11,12^ who potentially have decreased gut maturity and integrity compared to infants born >1500 g. Utilizing days to full enteral volume as proxy for feeding tolerance, we also showed that the median days was unchanged but with a narrower interquartile range and tighter distribution after implementation. This finding could reflect adherence to the feeding protocol itself, but we expect that patterns of feeding intolerance would have prolonged the time to attaining full feeding volume and yielded more variability, which we did not detect. Collectively, these data support exploring faster rates of standardized feeding advancement (>30 mL/kg/day) for preterm infants >1500 g to reduce dependency on vascular access. Exclusive early enteral nutrition has been demonstrated by Razzaghy et al. and is being investigated further in the larger FEED1 clinical trial.^40,41^

Feeding substrate is another important aspect to consider in feeding protocols. The limited number of studies on feeding protocol implementation that included larger infants all reported some degree of formula use.^35–37^ These studies predated the growing availability of DBM as supplementation to MBM.^17^ This is an important consideration as human milk is associated with both improved feeding tolerance and a decreased incidence of NEC.^42–44^ Feeding tolerance is likely influenced by human milk components that enhance maturation of the gastrointestinal tract and improve gut motility.^45,46^ Additionally, whey proteins, which are easier to digest than casein, are found in higher proportion in human milk compared to formula.^47^ Many of these benefits of unpasteurized human milk have been extrapolated to DBM, and its use has expanded beyond the VLBW population without much published data on outcomes for these larger and higher gestational age infants.^16,48^ In our case, the expanded eligibility criteria for DBM was well-accepted by the unit, possibly due to preexisting practice creep of allowing DBM to be offered to some infants of higher gestational ages. Overall, we encountered minimal barriers and no adverse events. However, the availability of DBM was not associated with a change in rates of MBM provision at discharge or direct breastfeeding at discharge, adding to the conflicting findings reported in the literature.^20–23^ As DBM use continues to expand into even higher gestational age infants, it is prudent to weigh carefully the increased cost of providing larger feeding volumes of DBM and, given the intrinsic differences between DBM and MBM, which benefits of human milk remain applicable.

Suboptimal growth is consistently a concern associated with the use of DBM, though with appropriate fortification strategies, adequate growth has been demonstrated in VLBW infants.^5,18,19,49^ Here we present novel evidence that appropriate anthropometric velocities and trajectories were achieved after the introduction of DBM for infants born >1500 g. However, in examining DBM exposure more closely, DBM intake was associated with a slight decrease in weight velocity (−1 g/d) and change in weight z-score (−0.03) per 10% increase in DBM intake. It is unclear whether the magnitudes of these parameter estimates are clinically significant, and the impact of DBM on growth in this population warrants further investigation. Furthermore, it is important to note that 42–46% of our cohorts received higher fortification beyond 24 kcal/oz to achieve the reported growth velocities. We recognize that our unit’s neonatal dietitians are particularly sensitive to the detection of growth faltering and liberally increase the fortification density in response to suboptimal growth. Due to the decreased macronutrient content of DBM, it is possible that DBM use in this population may also benefit from strategies beyond standard 24 kcal/oz fortification in order to meet nutritional goals.^50^ This may be an important aspect to consider when evaluating the generalizability of our results to other NICUs.

One major limitation to our study is that the >1500 g feeding protocol was implemented at the same time as the expanded DBM eligibility criteria, thus making it difficult to tease out their individual effect on feeding tolerance as an outcome. In the SIFT trial, there was a small degree of statistical interaction between the feeding substrate (human milk, formula, or a mixture) and the rate of feeding advancement, and the authors speculated that different diets may have unique risk-benefit profiles with regards to feeding strategies.^11^ We advocate that both a standardized feeding approach and the availability of DBM are valuable to patient outcomes and family satisfaction, and we have demonstrated they are safe to implement collectively for infants born >1500 g. Another major limitation is that the convenience sampling for patient selection based on cohort eras precluded adequate power calculations. Other limitations include the retrospective nature of our study, the inconsistent use of a recumbent measuring board for obtaining weekly length, and the focus on short-term outcomes. We also did not include late preterm infants, limiting the generalizability of our findings to very preterm and moderate preterm infants. The only additional clinical nutritional change occurred during the middle of the pre-implementation era: the upper cutoff for initiating dextrose infusions containing amino acids was increased from a birth weight of 1750–2500 g.

In summary, implementation of a feeding protocol with standardized volume advancements and DBM use may be associated with a more consistent time to achieving full enteral volume without impacting incidence of NEC in infants born >1500 g. Further prospective studies evaluating feeding practices, including faster feeding advancement rates, are warranted for this population. With monitoring and fortification, appropriate growth is possible with DBM use in non-VLBW preterm infants but needs additional adequately powered investigation.

## Supplementary Information

Appendix

## Data Availability

The datasets generated during and/or analyzed during the current study are available from the corresponding author on reasonable request.
