# Supplementary material for: Standardizing feeding strategies for preterm infants born greater than 1500 grams
Source: Pediatr Res. 2024 Aug 17;97(2):671–7. doi: 10.1038/s41390-024-03483-y (PMC12015110; doi:10.1038/s41390-024-03483-y)
Supplement: Supplementary file 1 — Appendix [file 41390_2024_3483_MOESM1_ESM.pdf]

Appendix 1: Standardized Feeding Protocol for Infants >1500 Grams and <34 Weeks

Parenteral nutrition (dextrose 10% with 3.8% trophamine (D10aa) on admission, then peripheral parenteral nutrition (PPN) or D10aa with ¼ normal saline (NS)) is initiated at 80 mL/kg/day and maintained at this volume with introduction of enteral feeding until a total volume of 160 mL/kg/day is reached, at which point fluid volume is weaned daily to maintain this total volume goal until fluids are discontinued.

Goal initiation of enteral feeding is within the first 24 hours of life if maternal milk is available. If maternal milk is not available at 24 hours of life and infant is <33 weeks, donor milk is offered if families give clinical consent; otherwise, Similac Special Care 24 kcal/oz is utilized.

Enteral feeding volume starts at 20 mL/kg/d for 24 hours, then increases by 15 mL/kg/day every 12 hours until 110 mL/kg/d. Human milk is fortified to 24 kcal/oz, and IV fluids are discontinued at this step, after which feeding volume advances by 10 mL/kg/d every 12 hours until 160 mL/kg/d is achieved. Birth weight is used for fluid calculations until 160 mL/kg/day is achieved, then current weight is used to continue to advance feeding volume as needed. DBM is transitioned to formula (either Similar Special Care or Neosure) at approximately 30 days or when infant's oral feeding intake is adequate for discharge preparation.

Example of a generated feeding protocol for an infant born at 1800 grams on July 1st.

Day of Enteral Feeding	Date	Time	Standard Volume	Actual Feeding Volume	# Feedings at this Volume	Approximate Caloric Density (Human Milk)	IV Fluid & Rate (mL/kg/day)	Total Fluid Goal
1	7/2	9:00 AM	**20 mL/kg**	**5 mL**	**x8**	**20 kcal/oz**	PPN 80 mL/kg/day	100 mL/kg/day
2	7/3	9:00 AM	**35 mL/kg**	**8 mL**	**x4**	**20 kcal/oz**	PPN 80 mL/kg/day	130 mL/kg/day
	7/3	9:00 PM	**50 mL/kg**	**11 mL**	**x4**			
3	7/4	9:00 AM	**65 mL/kg**	**15 mL**	**x4**	**20 kcal/oz**	PPN 80 mL/kg/day	160 mL/kg/day
	7/4	9:00 PM	**80 mL/kg**	**18 mL**	**x4**			
4	7/5	9:00 AM	**95 mL/kg**	**21 mL**	**x4**	**20 kcal/oz**	D10aa ¼ NS 50 mL/kg/day	160 mL/kg/day
	7/5	9:00 PM	**110 mL/kg**	**25 mL**	**x4**			
5	7/6	9:00 AM	**110 mL/kg**	**25 mL**	**x8**	**24 kcal/oz**	Let IV fluids run out after fortification	
6	7/7	9:00 AM	**120 mL/kg**	**27 mL**	**x4**	**24 kcal/oz**		
	7/7	9:00 PM	**130 mL/kg**	**29 mL**	**x4**			
7	7/8	9:00 AM	**140 mL/kg**	**32 mL**	**x4**	**24 kcal/oz**		
	7/8	9:00 PM	**150 mL/kg**	**34 mL**	**x4**			
8	7/9	9:00 AM	**160 mL/kg**	**36 mL**	**x8**	**24 kcal/oz**		

Supplemental Table 1 – Standardized feeding protocols for preterm infants born less than and greater than 1500 g

<table>
<tr><th rowspan="2"></th><th rowspan="2">Infants Born <1500 g (VLBW Protocol)</th><th colspan="2">Infants Born >1500 g and <34 weeks</th></tr>
<tr><th>Before Implementation</th><th>After Implementation</th></tr>
<tr><td>Trophics volume</td><td>15 mL/kg/d</td><td rowspan="8">Per clinical team discretion</td><td>20 ml/kg/d</td></tr>
<tr><td>Trophics timing (if clinically stable)</td><td>Within 36 hours of birth</td><td>Within 24 hours of birth</td></tr>
<tr><td>Feeding advancement increment before human milk fortification</td><td>10 mL/kg/d every 12 hours</td><td>15 mL/kg/d every 12 hours</td></tr>
<tr><td>Fortification to 24 kcal/oz[a]</td><td>At 75 mL/kg/d</td><td>At 110 mL/kg/d</td></tr>
<tr><td>Feeding advancement increment after human milk fortification</td><td>10 mL/kg/d every 12 hours (unchanged)</td><td>10 mL/kg/d every 12 hours</td></tr>
<tr><td>Enteral feeding volume when discontinuing parenteral nutrition or fluids</td><td>120-130 mL/kg/d</td><td>110 mL/kg/d</td></tr>
<tr><td>Typical enteral feeding volume goal</td><td>160 mL/kg/d</td><td>160 mL/kg/d</td></tr>
<tr><td>PICC for parenteral nutrition</td><td>Yes</td><td>No</td></tr>
<tr><td>Donor milk eligibility</td><td>Yes</td><td>No, with rare exceptions per attending discretion</td><td>For infants born <33 weeks</td></tr>
</table>

[a] In one step, using Similac Human Milk Fortifier Extensively Hydrolyzed Protein Concentrated Liquid (Abbott Nutrition, Chicago, IL, USA)

Supplemental Table 2 – Characteristics of infants fed per very low birth weight protocol, before and after implementation of >1500 g feeding protocol

	**Pre (n=10)**	**Post (n=18)**	**p-value**
Birth weight (g)	1573.0 (1518.1-1617.9)	1609.0 (1549.9-1708.1)	0.21
Gestational age at birth (weeks)	30.3 (29.1-31.6)	29.8 (29.3-30.7)	0.63
Clinical rationale for slower feeding advancements			
Suspect actual birth weight <1500 g	1 (10%)	0 (0%)	0.36
Birth weight 1500-1600 g	7 (70%)	9 (50%)	0.43
Gestational age <30 weeks	4 (40%)	9 (50%)	0.71
Twin was very low birth weight	1 (10%)	7 (39%)	0.19
Unclear rationale	0 (0%)	2 (11%)	0.52

Median (interquartile range) or n (%)
